# Protocol of a randomized controlled trial to investigate the efficacy and neural correlates of mindfulness-based habit reversal training in children with Tourette syndrome

**DOI:** 10.3389/fpsyt.2022.938103

**Published:** 2022-11-21

**Authors:** Yanlin Li, Junjuan Yan, Linyu Cui, Jiahui Chu, Xianbin Wang, Xi Huang, Ying Li, Yonghua Cui

**Affiliations:** ^1^Department of Psychiatry, Beijing Children's Hospital, Capital Medical University, National Center for Children's Health, Beijing, China; ^2^Cloud Services Innovation Laboratory, Institute of Intelligent Science and Technology, China Electronics Technology Group Corporation, Beijing, China

**Keywords:** Tourette syndrome, habit reversal training, mindfulness, protocol, randomized control trial

## Abstract

**Background:**

Tourette syndrome (TS) is a developmental neuropsychiatric disorder. Behavior therapy, especially habit reversal training (HRT), has gradually become regarded as one of the core therapies for TS. Mindfulness approaches can improve psychological adjustment and reduce stress and anxiety, suggesting potential benefits when incorporated into behavior therapy. To improve the efficacy of HRT, we combined it with mindfulness, an approach named mindfulness-based habitual reversal training (MHRT). The aim of this protocol is to investigate the efficacy and neural mechanisms of MHRT for TS.

**Methods/design:**

We will perform a randomized control trial (RCT) to evaluate the efficacy and neural mechanisms of MHRT. The sample will include 160 participants (including 120 patients with TS and 40 healthy controls). The patient sample will be randomly divided into three groups exposed to three different types of training: MHRT, HRT, and psychoeducation and supportive therapy (PST). Participants will be assessed and undergo resting-state fMRI scans at baseline and at the end of the 12-week training. The Yale Global Tic Severity Scale (YGTSS) and Premonitory Urge for Tic Scale (PUTS) will be used to assess the severity of tic symptoms and premonitory urges. The primary outcomes are change scores on the YGTSS and other assessments from baseline and the end of the training. The secondary outcomes are the neural correlates of these trainings among these groups based on graph theory, which is used to characterize brain functional connectivity networks. The default mode network (DMN) and the salience network (SN) will be assessed (which have been associated with mindfulness as well as the generation of tic symptoms) by network parameters, including clustering coefficients and shortest path lengths. Changes in these network parameters will be regarded as the neural correlates of the behavioral training.

**Discussion:**

MHRT was newly developed for the treatment of TS. MHRT may lead to greater reductions in tic severity than traditional HRT. Changes in the network parameters of the DMN and SN may show associations with the efficacy of MHRT.

**Clinical trial registration:**

http://www.chictr.org.cn, ChiCTR2100053077, China.

## Introduction

Tic disorders (TDs) are mainly first recognized in children and adolescents (1). They are neurodevelopmental disorders characterized by motor and/or phonic tics. TDs are graded into three categories according to clinical manifestation: transient TD, chronic TD (motor or vocal), and Tourette syndrome (TS). TS is characterized by the presence of both movements and vocalizations, which last for more than 1 year (2). TS is a common disorder in children and adolescents, with a prevalence of 1 and 3% (3), respectively. TS has been regarded as the most serious and intractable type of TD. Previous studies have estimated that ~60–80% of symptoms of TS can last beyond the age of 16, and ~23% of these individuals will be left with a moderate and severe tic that causes serious social impairments (4). The cause of TS has not yet been fully elucidated, and the treatment also lacks definitive and effective methods. Therefore, studies of the pathogenesis and therapeutics for TS show great clinical significance and need to be further developed.

At present, the treatment of TD mainly includes psychological interventions and pharmacological treatment. Medications (e.g., aripiprazole and tiapride hydrochloride) are the main form of treatment and have been relatively successful in treating symptoms. However, drug treatment has some side effects (e.g., weight gain and extrapyramidal syndrome) (5). Consequently, nondrug treatments [e.g., habit reversal training (HRT) and transcranial magnetic stimulation], especially behavior therapy (mainly HRT), have gradually attracted attention due to their safety (6).

Among many behavioral therapies for TD, the most pivotal and classic therapy is HRT. The latest guidelines from the American Academy of Neurology in 2019 also suggested HRT as the first-line treatment for TD (7). Moreover, behavior therapy has been recommended in Europe and Canada as a first-line treatment for TD (8–10). Much experimental evidence to date has shown the safety and effectiveness of behavior therapy (6, 11–13). The long-term effects of behavior therapy for tics also support guidelines recommending behavior therapy as the first-line intervention for tics (14, 15). Research on behavior therapy has implications not only for clinical practice but also for an understanding of the etiology of TD. For example, McGuire et al. found that cognitive control processes may influence the tic severity reductions observed with behavior therapy (16).

However, based on some outcome studies, the efficacy of HRT still needs to be further investigated. A randomized control trial (RCT), in a comparison trial of HRT to exposure and response prevention (ERP), had results indicating that the ERP group experienced significantly greater reductions in tic severity (17). In another comparison trial, HRT and awareness training were compared, and participants in the HRT group experienced only a minimal benefit above awareness training (18). These studies showed that awareness training is an important component of HRT. Moreover, the treatment effects are also typically affected by age. Participants under the age of 10 may have difficulty learning awareness training due to difficulty with premonitory urge recognition (19). The key step of HRT is the detection of premonitory urges (PUs) (20), but insufficient awareness may lead to decreased curative effects, especially in younger individuals. Moreover, tics may be accompanied by emotional problems, such as anxiety and depression, but traditional HRT training does not adequately resolve these emotional problems. More improvements are needed to compensate for the limitations of HRT.

Mindfulness is attention to present moment experience without judgment, primarily to become reflectively aware of the nature of emotional and cognitive patterns based on a shift of emphasis from contents (e.g., of perceptions or thoughts) to processes (21). Mindfulness training shows salutary effects on cognition (increased awareness) and emotional wellbeing (improved mood and stress relief) in children and adolescents (22–24), which addresses the two problems of HRT. Therefore, we will attempt to integrate mindfulness into HRT and call the new therapy “mindfulness-based habitual reversal training” (MHRT). Improvements in the curative effects may be mainly based on two links: the first link is that mindfulness can enhance awareness of PUs; the second link is that mindfulness can alleviate the anxiety associated with tics during relaxation training (25).

Some theoretical understandings of the brain mechanisms involved in mindfulness take their lead from considerations about the roles of the major brain networks and their interplay (21). Functional connectivity in intrinsic brain networks, which include the salience network (SN), default mode network (DMN) and central executive network (CEN), has been suggested to be important in the networks prominently involved in cognition and mindfulness (26, 27). Mindfulness training involves participants training the attention system to inhibit irrelevant sensory stimuli and enhance relevant sensory stimuli (28) and consists of focusing attention on physical sensations (29). This suggests that mindfulness can improve the patient's perception of PUs. Mindfulness-mediated decreased connectivity between the cuneus and SN may be related to self-awareness (27). The primary node of the SN is within the anterior insular cortex (30). Neuroimaging studies have suggested that PUs are associated with the insula and anterior cingulate cortex (ACC) (31, 32). A review indicated that mindfulness-based treatments may be effective in restoring connectivity between the DMN and the CEN and SN in individuals with PTSD (33). Meditation practitioners showed significantly stronger connectivity between the DMN and SN (34), as well as increased connectivity in systems important for emotion and reward (35). This may explain why mindfulness reduces anxiety and depression and enhances wellbeing. On the other hand, TS patients exhibited a reduction in serial information transfer within the DMN and SN (36). TD patients benefit from increased functional connectivity within the DMN as a compensatory function (37). From the perspective of brain networks, we speculate that mindfulness can provide useful support for conventional HRT in TS patients. To understand the underlying mechanisms of mindfulness in TS, we will focus on two key brain networks, the DMN and SN.

Therefore, this project plans to carry out an RCT of MHRT in the treatment of TS, further systematically verify its efficacy and explore the brain mechanisms underlying the synergy of MHRT. The significance and value of this study are to form a suitable psychological and behavioral intervention scheme for TS, resolve the dilemmas in clinical treatment to a certain extent, and provide data for further clarifying the pathophysiological mechanisms underlying TS.

## Methods/design

### Participants

#### Recruitment of participants

Recruitment of TS patients will be from the Department of Psychiatry, Beijing Children's Hospital, where participants will have received a diagnosis of TS from an experienced multidisciplinary team including at least two attending psychiatrists using the diagnostic criteria in the DSM-5. The inclusion criteria were as follows: (1) 7–12 years old; (2) Chinese children's Wechsler intelligence test IQ ≥ 80; (3) first onset with no previous treatment; and (4) right-handed. The exclusion criteria were as follows: (1) diagnosed with other mental disorders, apart from obsessive–compulsive disorder (OCD) or attention deficit hyperactivity disorder (ADHD); (2) diagnosed with epilepsy or other neurological diseases (including craniocerebral trauma); (3) claustrophobia; and (4) metallic foreign objects in the body. The recruitment period was predefined for one and a half years (May 2022 to Dec 2023).

Recruitment of healthy controls will be from a primary school in Beijing. The inclusion criteria were as follows: (1) aged 7–12 years; (2) IQ ≥ 80; and (3) right-handed. The exclusion criteria were as follows: (1) diagnosed with neurological or mental diseases; (2) claustrophobia; and (3) metallic foreign objects in the body.

#### Sample size estimation

In accordance with the experimental design, three groups of participants will be included: the HRT group, MHRT group, and psychological education and supportive therapy (PST) group.

In addition, for the sample size, we use the formulas (see Supplementary Figure S1) to **calculate** the sample size (α = 0.05, 1–β = 0.8). Taking YGTSS scores as the indicator of a curative effect and based on the effect size of 0.68 calculated in a preliminary pilot test, it was calculated that at least 23 participants were needed in each group, and the drop-out rate was estimated to be 20%. Based on previous experience, 15% of MRI scans have invalid data, resulting in at least 34 subjects being required in each group (38). Therefore, 40 patients will be recruited in each group, for a total of 120 patients in this study. In addition, another 40 healthy controls will be recruited as blank controls for the behavioral and imaging research. A total of 160 participants will be included.

All evaluators will be blinded, and the treatment condition assignment will not be disclosed until after the intervention. The clinical evaluator will not be informed of which therapeutic modality the participants will receive in the evaluation process. Moreover, all dropouts must be accounted for and reported. The reasons for dropouts are mentioned. Intention-to-treat analysis was used to assess the missing data.

### Assessment instruments

#### Schedule for affective disorders and schizophrenia for school-age children-lifetime version (Kiddie-SADS-L)

The Kiddie-SADS-L can assess the current and history of mental disorders in children and adolescents, including behavioral disorders such as attention-deficit/hyperactivity disorder, opposition defiance disorder, and conduct disorder, as well as common psychopathological phenomena such as affective disorder, anxiety disorder, and psychotic disorder (39). It has content validity, the interrater consistency was 93–100%, and the test-retest reliability coefficient for common mental disorders ranged from 0.63 to 1.00 (40).

#### Yale global tic severity scale

The YGTSS was developed based on the Tourette Syndrome Global Scale and displayed very good internal consistency, interrater reliability, and convergent and divergent validity (41). Its total (motor + phonic) tic severity score can identify clinically meaningful exacerbations of tics. The YGTSS is the only scale for which cutoff values of score changes indicate clinically relevant exacerbations and treatment responses, making it the most suitable instrument for prospective follow-up in clinical observational longitudinal studies and therapeutic trials.

#### Premonitory urge for tic scale

The PUTS has been developed and has shown good psychometric properties (19). While this scale has been widely used in clinical research related to tics, the PUTS reports the feelings before tics and the corresponding severity but does not include the locations of PUs (42).

#### Children's Yale-Brown Obsessive-Compulsive scale

The CY-BOCS, designed for children, is a modified version of the Yale-Brown Obsessive-Compulsive Scale (Y-BOCS) (43). The CY-BOCS is a clinician-rated, semistructured instrument designed to assess the symptom severity of obsessions and compulsions. Each item is rated on a 5-point ordinal scale: 0 = none, 1 = mild, 2 = moderate, 3 = severe, and 4 = extreme. The total score is obtained by adding the scores for Items 1 through 10 (range = 0–40). The subscales of obsessions (Items 1 through 5) and compulsions (Items 6 through 10) each range from 0 to 20. A considerable body of data from around the world attests to the reliability and validity of the instrument in children with OCD. The adult version (Y-BOCS) has been translated into Chinese, with a Cronbach coefficient of 0.75 (44). However, evidence of the reliability and validity of the CY-BOCS in Chinese children and adolescents is lacking. In our previous study, we used it as a tool for TS evaluation. We included 200 samples, and the Klezbach coefficient of the scale in children with tic symptoms was more than 0.85.

#### Clinical global impression

The CGI provided a brief, stand-alone assessment of the psychiatrist's view of the patient's global functioning before and after initiating study treatment (45). This scale mostly assesses the severity of psychopathology and changes from the initiation of treatment, resulting in an efficacy index.

#### Five facet mindfulness questionnaire

The FFMQ is a 39-item self-report measure (46) that is used to evaluate the internal perception of self-report to clarify the changes in subjective perception. The items on this scale focus on self-awareness of internal feelings, such as “when I walk, I deliberately pay attention to the feelings in physical movement”; or focus on emotions, such as “I focus on how my emotions affect my thoughts and behavior”.

#### Screening child anxiety-related emotional disorders

The SCARED is a self-reported measurement tool specifically used to measure anxiety symptoms of children and adolescents, including the five factors of somatic/panic disorder, generalized anxiety disorder, separation anxiety disorder, social phobia, and school phobia, with a total of 41 items (47). The participants rated the frequency of each symptom on a 3-point scale of 0–2, and the average score of the relevant items was calculated as the specific anxiety symptom score and total anxiety score. Previous studies have shown that the scale has been widely used in the screening of children's anxiety and has good reliability and validity in the context of Chinese culture (48).

#### Wechsler intelligence scale

The Chinese version of the Wechsler Intelligence Scale for children (49) was used to evaluate the participants' intelligence quotient (IQ). The scale has a national norm. It is divided into urban versions and rural versions. The split-half reliability of each subtest score is high (the split-half correlation coefficient is ~0.8), the IQ split-half reliability is above 0.9, and the test-retest reliability range is 0.59–0.86 (50). It has also been shown to have structural validity. In China, this is an important tool for assessing children's IQ level.

### Assessment procedures

**Figure 1:**
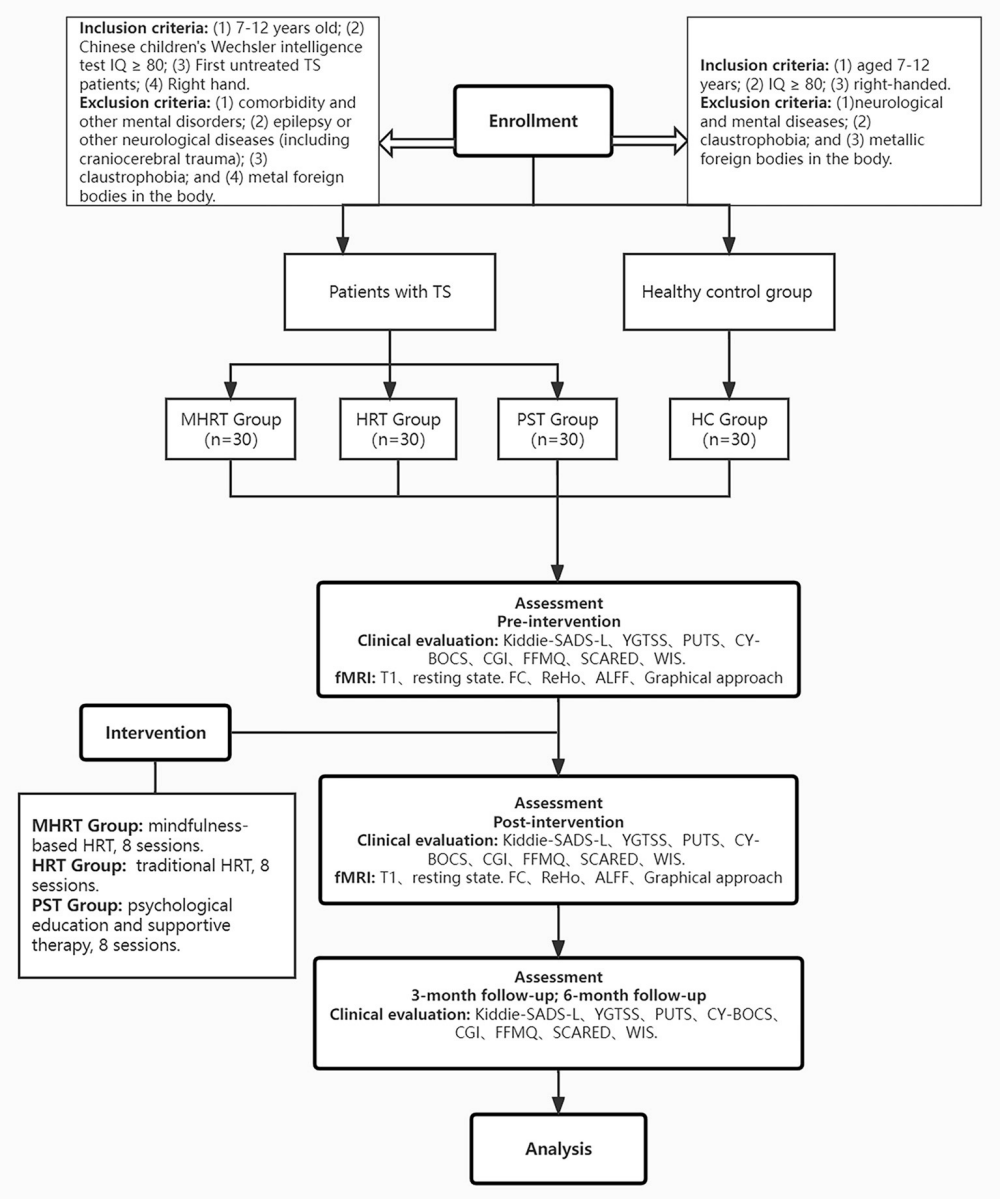
Proposed procedure and flow of participants of the study. IQ, Intelligence quotient; TS, Tourette syndrome; MHRT, Mindfulness-based habitual reversal training; HRT, Habit reversal training; PST, psychoeducation and supportive therapy; HC, healthy controls; Kiddie-SADS-L, Schedule for Affective Disorders and Schizophrenia for School-Age Children-Lifetime Version; YGTSS, Yale Global Tic Severity Scale; PUTS, Premonitory Urge for Tic Scale; CY-BOCS, Children's Yale-Brown Obsessive-Compulsive Scale; CGI, Clinical Global Impression; FFMQ, Five Facet Mindfulness Questionnaire; SCARED, Screening child anxiety-related emotional disorders; WIS, Wechsler Intelligence Scale.

The clinical evaluation process will mainly be completed by two attending child psychiatrists. Before the formal evaluation, an evaluation of the consistency between raters will be completed. The clinical evaluation will be carried out only after the consistency of the raters is above 0.85. The time points for the evaluation mainly included the baseline test, within 1 week after the completion of eight sessions of intervention, and at the end of 1 month after eight sessions of intervention. The patients will be followed up at the end of 3 and 6 months after the eight sessions of intervention. For more details, see Figure 1.

This study is a single-blind RCT. Each child was randomized to either an MHRT, HRT or PST. All groups were described to participants as active interventions with potential but unknown benefits. Assessors were blinded to the treatment conditions.

### Interventions for the different groups

**Figure 2:**
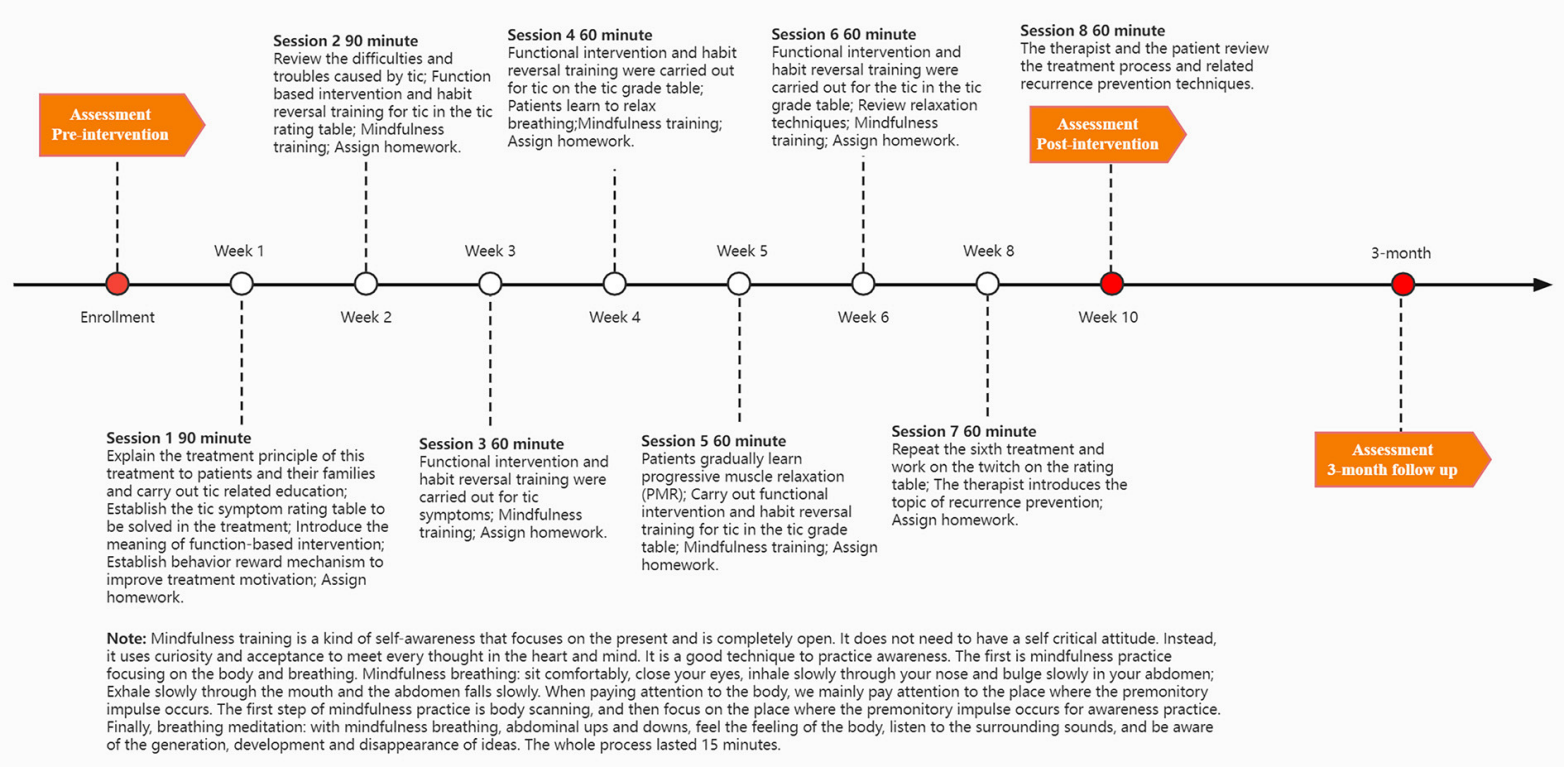
MHRT and HRT Process. Only the MHRT group received mindfulness training.

There are three main types of intervention techniques used in this study: traditional HRT, MHRT, and PST. The intervention includes a total of eight sessions, and a consistent time frame will be used with each intervention method. More details can be seen in Figure 2.

The kind of mindfulness meditation used in the clinical setting is a secularized amalgamation of Buddhist contemplative practices (51). Mindfulness meditation is given during all sessions. The content is focused on learning how to mindfully attend to body sensations, especially PUs. Mindfulness training includes mindfulness of the breath, body scanning, sitting meditation, and mindful movement in the consulting room. The whole process lasted 15 min in each session. Additionally, the training recommends that participants apply these mindful practices in daily life. Mindfulness modules are applied to aid participants in recognizing PUs with the ultimate effect of being able to engage in competing responses in a more accessible manner. Details on the components of the mindfulness training are provided in Table 1.

**Table 1:** Content of mindfulness training in MHRT.

**Session**	**Thematic curriculum**	**Mindfulness modules**
1	Understand mindfulness	1. What is mindfulness; 2. How can mindfulness be useful for addressing tic symptoms; 3. Body scan; 4. Homework: pausing during daily life.
2	Be in the present	1. Be in the present; 2. Tic and unpleasant events journal; 3. Mindful breathing; 4. Homework: mindful breathing and mindful walking in daily life.
3	Be mindful of PUs	1. Recognize the PUs and tic; 2. Mindful breathing; 3. Mindful stretching; 4. Homework: 3-min breathing space in daily life.
4	Accept difficulties in Competing Response Training	1. Difficulties journal; 2. Learn to accept difficulties; Mindful meditation (be with difficulties); 3. Homework: 3-min breathing space in daily life
5	Emotions and stress responses	1. Recognize mood without judgment; 2. Let thoughts about mood be; 3. Mindful meditation (be with thoughts about mood); 4. Homework: pausing during daily life
6	Enjoy daily happiness	1. Be mindful of happiness; 2. Pleasant events journal; Mindful meditation (be with daily happiness); 3. Homework: 3-min breathing space in daily life
7	Discuss relapse prevention strategies	1. Feel PUs mindfully; 2. Be mindful of emotions; 3. Discussion relapse prevention strategies; 4. Homework: 3-min breathing space in daily life.
8	Continued mindfulness practice	1. Discussion on awareness of PUs and emotions; 2. Consider continued mindfulness practice.

PUs, premonitory urges; MHRT, Mindfulness-based habitual reversal training.

Traditional HRT includes general awareness training, competitive response training, relaxation training, and homework (20). Although traditional HRT includes relaxation techniques, diaphragmatic breathing exercises and progressive muscle relaxation, there are also key differences between MHRT and traditional HRT. These differences are reflected in practice time and content.

PST is a relatively basic treatment among psychological and behavioral interventions for TS (52) and mainly includes two aspects. First, parents are trained in topics including popular scientific perspectives of the disease, how to analyze factors that induce exacerbation of tic symptoms, and how to encourage children to deal with the disease for 20–30 min. The second aspect involves communication and interactions with the children. The conversation with the children lasted for more than 30 min. The main contents include the feeling and understanding of tics, helping children deal with accompanying emotions, and evaluating and addressing shame and stigma. The intervention times were consistent with the other two therapies.

### MRI scanning

#### Basic information for MRI scanning

Brain imaging data from all participants will be acquired using a 3.0 T scanner (MR-750, General Electric, USA). Before data collection, participants will become familiar with the scanning environment. Then, participants are placed supine, asked to remain awake, and placed into the scanner. Head motion will be minimized by inserting special-purpose foam pads. During this process, the participants needed to keep their eyes closed and their bodies motionless.

#### MRI scanning sequence

T1-weighted images will be acquired using a sequence with the following parameters: repetition time (TR) = 7 ms, echo time (TE) = 3.2 ms, flip angle = 8 degrees, resolution = 240 × 240, slices = 192, and slice thickness = 1 mm. Functional whole-brain images will be acquired using an echo planar imaging sequence (TR = 2,280 ms, TE = 30 ms, image matrix = 64 × 64, FOV = 224 mm, flip angle = 80°, slice thickness = 3 mm, distance factor = 17%, voxel size = 3.5 × 3.5 × 3.0 mm, and 36 axial slices). A total of 80 images aligned to PC–AC will be acquired per functional imaging block.

#### Preprocessing

Processing steps will use MATLAB and Data Processing Assistant for Resting State fMRI (DPARSF) (53). The first five volumes will be discarded to allow the magnetization to approach a dynamic equilibrium and for the subjects to get used to the scanner noise. Data preprocessing, including slice timing, head motion correction (a least squares approach and a 6-parameter spatial transformation) and spatial normalization to the Montreal Neurological Institute (MNI) template (resampling eight voxel sizes of 3 × 3 × 3 mm), will be performed. A spatial filter of 4-mm FWHM (full-width at half maximum) will be used. Participants showing head motion above 3.5 mm of maximal translation (in any direction of x, y or z) and 1.0° of maximal rotation throughout the course of the scan will rule out subsequent statistical analysis.

#### Construction of the network

The template of the neuroimaging research laboratory of Stanford University (Functional Imaging in Neuropsychiatric Disorders Lab website; http://findlab.stanford.edu/functional_ROIs.html) will be used to explore the DMN and SN (54). For the ROIs of the DMN and SN, see Supplementary Tables S1, S2.

We will use the Brain Connectivity Toolbox to extract the connection information between various brain regions and draw the connection diagram at the macro level. This will be used to quantitatively describe the structure, function, connection model and topological characteristics of the human brain network. We will mainly focus on the brain regions related to PUs: the insula, ACC, supplementary motor area (SMA) and prefrontal cortex. We will take the DMN and SN as the main brain function networks to consider.

Degree, global efficiency, local efficiency, path lengths and cluster coefficients will be used to investigate the relationships between the changes in these two brain functional networks and the improvements in tics and PUs and to clarify the possible brain mechanisms underlying the effects of MHRT from the perspective of brain functional networks. We will assess global network integration, segregation and connectivity between modules using the following measures for the DMN and SN: (1) degree: number of edges connected to a node; (2) global efficiency: global transmission capacity of the network; (3) cluster coefficient: degree of network collectivization; (4) local efficiency: nodal connections to within-network nodes; (5) path length: optimal path from one node to another. To assess the reliability of the results and enhance comparability with previous research, exploratory analyses on the absolute Fisher r-to-z transformed Pearson correlation connectivity matrices will be performed using the same graph indices. The calculation formulas for these parameters are presented in Supplementary Figure S2.

### Statistical analysis

Measures will be analyzed using IBM SPSS Statistics version 22 (IBM, Armonk, NY, United States).

First, the efficacy of MHRT will be tested using repeated measures analysis of variance (ANOVA). A 3 × 3 mixed design will be used to examine the main effects of group (MHRT, HRT or PST) as a between-subjects factor and time point as a within-subjects factor, as well as interaction effects. Second, for the imaging study, we will assess connectivity between the DMN and SN and compute the average Fisher r-to-z transformed Pearson correlations of the resting-state fMRI time-series between the nodes that belong to these resting-state networks. Pearson correlations will be used to replicate earlier functional connectivity studies. For analysis of the abovementioned neuroimaging indices, we will use univariate mixed-model analyses to assess the effects of MHRT and HRT. We will use the after-training outcomes as dependent variables, the group (MHRT, HRT, PST, and HC) as the independent variable and the baseline measure of the outcome as a covariate. We will also perform these analyses correcting for age, years of education and sex.

Measurement data will be presented as the mean ± standard deviation (x ± s). The demographic and efficacy indices of the three intervention groups before and after the intervention will be compared by multifactor analysis of variance (measurement data), and repeated measures analysis of variance and paired *t*-tests will be used for comparisons between multiple groups and before and after the intervention. In addition, we regarded at least 30% changes in YGTSS scores $\left[(\frac{\mathrm{Pr}e_{\left(YGTSS\right)}-Post_{\left(YGTSS\right)}}{\mathrm{Pr}e_{\left(YGTSS\right)}}\right)\times 100\%]$ as the clinically meaningful level.

For the analysis of global graph measures, we consider a significance level of α = 0.05 significant. To correct for multiple comparisons in subnetwork analyses, we will use a D/AP-Sidak adjustment that considers the mutual correlation between outcome measures (https://www.quantitativeskills.com/sisa/calculations/bonhlp.htm). For between-network connectivity analyses, we will compute an α corrected for three comparisons, i.e., adjusted for the correlation (*r* = 0.544) between the three indices after training (αBN = 0.031). For subnetwork graph analyses, we will compute a separate α for each graph measure adjusted for the correlation between the respective subnetwork measures at baseline (rFPN = 0.387, αFPN = 0.026; rDMN = 0.408, αDMN = 0.026; rSN = 0.413, αSN = 0.027).

### Clinical trial registry

The study is a double-blind RCT reviewed and approved by the Ethics Committee of Beijing Children's Hospital, Capital Medical University. All participants and their guardians will provide their informed consent and sign the informed consent form. If the study participants feel unwell, they can withdraw from the study at any time and have a follow-up doctor for further examination and treatment. The trial is registered on the Chinese Clinical Trial Registry (ChiCTR2100053077, http://www.chictr.org.cn).

## Discussion

This protocol describes a study assessing the efficacy of MHRT for children with TS and associated brain network mechanisms. To the best of our knowledge, this is an original intervention technology that may be valuable for the non-drug treatment of TS. The protocol was designed to investigate multiple aspects of the treatment response, not only tic severity but also the effects of MHRT, HRT and PST on psychosocial functioning and neural physiological mechanisms.

Our results may be consistent with previous studies in patients with TS that compared the effectiveness of HRT and PST in a similarly large sample of TS patients. Based on available pilot data, we predict that patients receiving MHRT or HRT may show significant and clinically meaningful reductions in tic severity compared with patients receiving PST. We speculate that augmenting HRT with mindfulness training will enhance tic severity reductions and result in stronger awareness and lower anxiety levels. The fMRI scans may show functional changes in brain networks associated with mindfulness.

Mindfulness training can improve performance in attention, working memory and cognitive control tasks (55–57). With the potential benefits of mindfulness in children with common neurodevelopmental disorders (58), more research has been conducted in recent years. In 2020, a study investigated the feasibility of a family-based mindfulness intervention in 100 attention-deficit/hyperactivity disorder (ADHD) children's families and found that the intervention group showed greater improvements in children's ADHD symptoms (59). In 2021, Muratori et al. in a 50-child sample of adolescents with ADHD and oppositional defiant disorders showed that participating in a mindfulness meditation was associated with a greater reduction in hyperactive behaviors in the school context (60). Another pilot study using mindfulness training for children aged 8–12 years with ADHD (*n* = 140) and their parents also showed promising results (61). A study in Hong Kong demonstrated the feasibility of a mindfulness program in the Chinese context (62). Accumulating evidence supports the applicability of mindfulness-based therapy for those with mental health and neurodevelopmental disorders. The theoretical basis suggests that it can also be beneficial for patients with TS.

The mechanisms underlying mindfulness mainly involve two brain networks: the DMN and SN (26, 63, 64). The insula and anterior cingulate, which are responsible for premonitory impulses, are the key nodes in the SN network, and this network is dysfunctional in those with TS (31, 32, 65). Research shows that the main function of this network is to screen internal and external “key stimuli” (22, 63). We speculate that with the integration of mindfulness training technology, the first improvement with MHRT is enhancing the brain functional connections of the network such that the neural signals of PUs can be more easily “screened”. The default network is more closely related to “self”-related processing, and mindfulness training can improve an individual's self-awareness (66). Therefore, improved brain function involving the default network may improve the ability to detect PUs. In addition, the relief provided by mindfulness for the emotions associated with anxiety will also contribute to the awareness of PUs.

We need to be aware of the importance of HRT and spur further development. Psychological studies have argued that the learned associations acquired in HRT disrupt the negative reinforcement cycle that maintains tic expression (67–70). A large amount of evidence-based medical evidence confirms the effectiveness of this process. However, as a treatment response is associated with a 25–35% reduction in tic severity (71), there is room for improvement in therapeutic outcomes. In addition, few hospitals have performed such therapy, and no efforts have been dedicated to the study and development of the corresponding therapy plan in China. Exploring the biological mechanism and selecting the synergistic therapeutic mode based on the mechanisms, appropriately simplifying treatment sessions, developing group and network therapy, and strengthening the cultivation of professional personnel can make this therapeutic service more effective. Therefore, we emphasize the urgency of the development of this therapy in China. This protocol provides practical clinical significance and value given the current situation. MHRT can play a synergistic role based on HRT, but its efficacy evaluation and elucidation of brain mechanisms have important implications. Through this RCT, we can determine how to best evaluate the most important link between HRT and MHRT interventions and PUs. From a more objective perspective of the brain network, we will clarify the synergistic mechanisms underlying the effects of MHRT. This can provide a more practical and effective psychological intervention for children with TS to train and practice.

## Limitations

The symptoms of tic disorder are characterized by volatility. Its severity will be affected by life events, emotions, physical diseases such as infection and so on. Such fluctuations will affect our judgment of the treatment effect. To reduce misjudgment, we will use a scale related to life events and emotions in the evaluation and establish a 3-month follow-up period.

To ensure the quality of and effects obtained with this RCT, we will adopt a one-on-one treatment protocol. Due to the lack of therapists, our experiment will take a long time. In this process, participants will be required to come to the clinic at a fixed time every week. This may also result in the loss of these school-age participants. Online training and group therapy may be ways to solve this problem in the future.

## Conclusion

The findings from this work will (1) provide evidence-based information on optimizing behavior therapy targeted at youth with TS. (2) The findings will shed light on the benefits of mindfulness in improving awareness and in reducing stress among children with TS. (3) The examination of brain networks will advance our understanding of the process underlying the pathogenesis of TS and how mindfulness works.

## Author contributions

YaL: writing-original draft. LC, JC, XW, and XH: section of analysis of network and interpretation of data. YiL and JY: writing-review and editing. YC: supervision. All authors contributed to the article and approved the submitted version.

## Conflict of interest

Author XH was employed by the company China Electronics Technology Group Corporation. The remaining authors declare that the research was conducted in the absence of any commercial or financial relationships that could be construed as a potential conflict of interest.

## Publisher's note

All claims expressed in this article are solely those of the authors and do not necessarily represent those of their affiliated organizations, or those of the publisher, the editors and the reviewers. Any product that may be evaluated in this article, or claim that may be made by its manufacturer, is not guaranteed or endorsed by the publisher.

## Supplementary material

Supplementary Figure S1 Formulas for the calculation of sample size.

Supplementary Figure S2 Formulas for several important parameters for the networks.

Supplementary Table S1 DMN ROI. SN, Salience network; ROI, Region of interest; MNI, Montreal Neurological Institute.

Supplementary Table S2 SN ROI. DMN, default mode network; ROI, region of interest; MNI, Montreal Neurological Institute.
