# Supplementary figures and images for: Protocol of a randomized controlled trial to investigate the efficacy and neural correlates of mindfulness-based habit reversal training in children with Tourette syndrome
Source: Front Psychiatry. 2022 Nov 21;13:938103. doi: 10.3389/fpsyt.2022.938103 (PMC9719972; doi:10.3389/fpsyt.2022.938103)

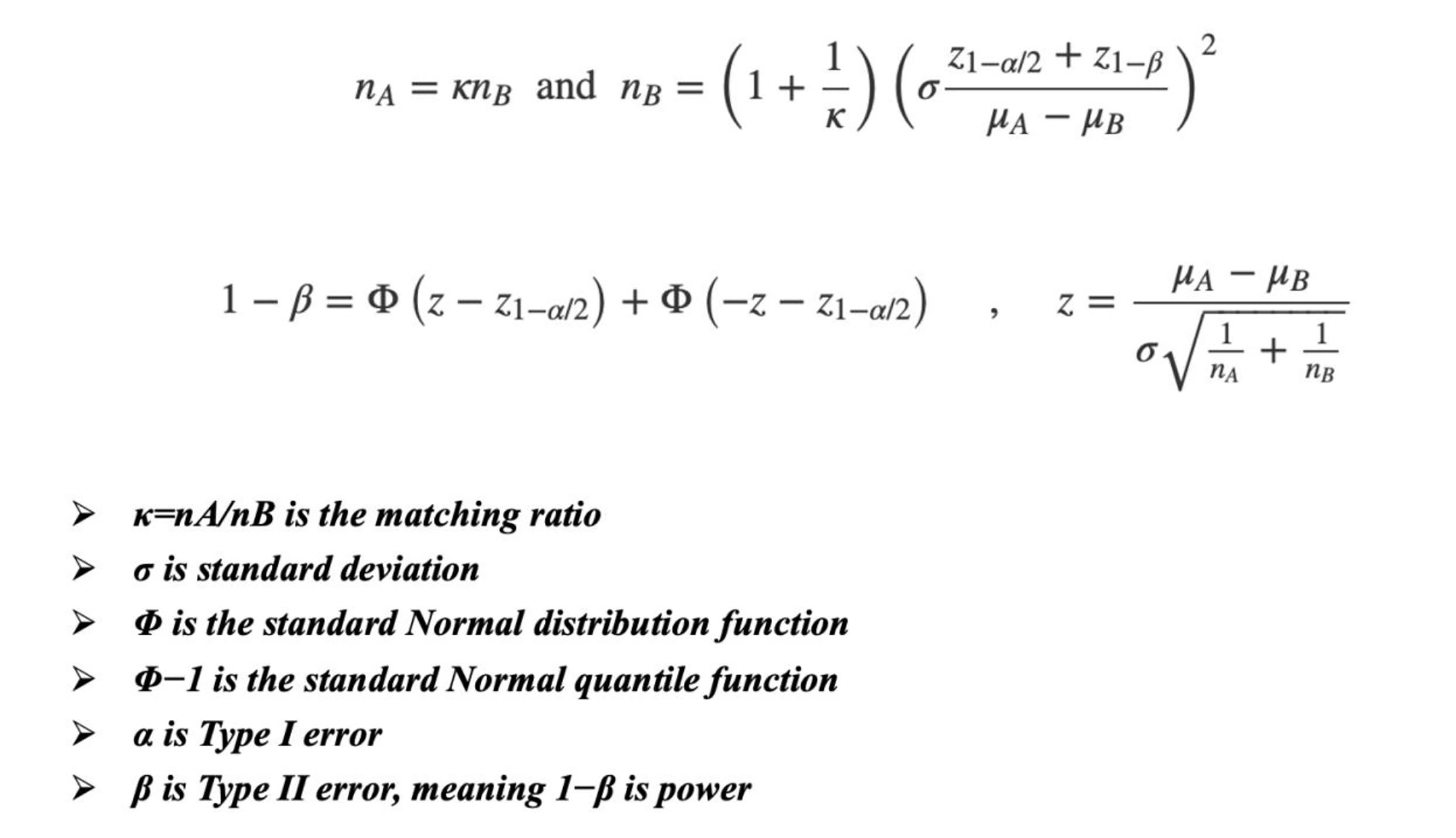

Supplement: Supplementary Figure S1 — Formulas for the calculation of sample size. [file Image_1.TIF]

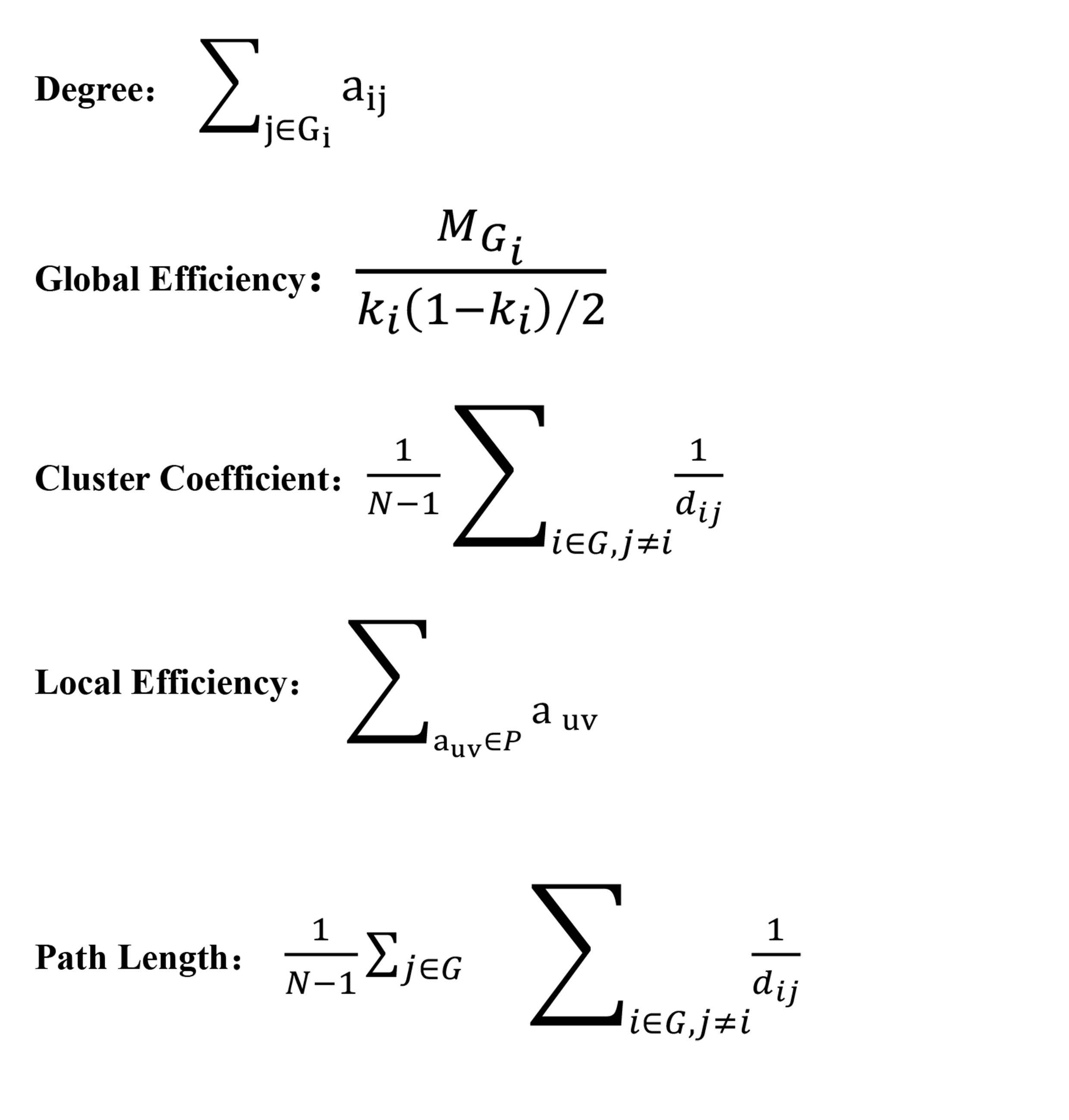

Supplement: Supplementary Figure S2 — Formulas for several important parameters for the networks. [file Image_2.TIF]
