# Supplementary material for: Protocol of a randomized controlled trial to investigate the efficacy and neural correlates of mindfulness-based habit reversal training in children with Tourette syndrome
Source: Front Psychiatry. 2022 Nov 21;13:938103. doi: 10.3389/fpsyt.2022.938103 (PMC9719972; doi:10.3389/fpsyt.2022.938103)
Supplement: Supplementary Table S1 — DMN ROI. SN, Salience network; ROI, Region of interest; MNI, Montreal Neurological Institute. [file Table_1.docx]

| DMN. ROIs | | Main area | MNI coordinates（X，Y，Z） | | |
| --- | --- | --- | --- | --- | --- |
| Dorsal DMN | ROI1 | Medial frontal gyrus | 0 | 49 | 12 |
|  | ROI2 | Left angular gyrus | -50 | -68 | 30 |
|  | ROI3 | Right superior frontal gyrus | 22 | 40S | 40 |
|  | ROI4 | Posterior cingulate cortex | 0 | -54 | 27 |
|  | ROI5 | Middle cingulate gyrus | 4 | -16 | 32 |
|  | ROI6 | Right angular gyrus | 52 | -64 | 28 |
|  | ROI7 | Thalamus | 0 | -12 | 5 |
|  | ROI8 | Left hippocampus | -24 | -28 | -17 |
|  | ROI9 | Right hippocampus | 26 | -21 | -17 |
| Ventral DMN | ROI10 | Left posterior Cingulate gyrus | -9 | -54 | 13 |
|  | ROI11 | Left middle frontal gyrus | -24 | 10 | 56 |
|  | ROI12 | Left fusiform gyrus | -28 | -30 | -19 |
|  | ROI13 | Left middle occipital gyrus | -36 | -82 | 31 |
|  | ROI14 | Posterior cingulate cortex | 14 | -52 | 13 |
|  | ROI15 | Precuneus | 0 | -60 | 54 |
|  | ROI16 | Right middle frontal gyrus | 24 | 32 | 42 |
|  | ROI17 | Right fusiform gyrus | 29 | -32 | -21 |
|  | ROI18 | Right middle occipital gyrus | 44 | -74 | 31 |
|  | ROI19 | Right cerebellum | 16 | -44 | -53 |

**Table S1. DMN ROI**

*Note: DMN, default mode network; ROI, region of interest; MNI, Montreal Neurological Institute.*
