# Supplementary material for: Protocol of a randomized controlled trial to investigate the efficacy and neural correlates of mindfulness-based habit reversal training in children with Tourette syndrome
Source: Front Psychiatry. 2022 Nov 21;13:938103. doi: 10.3389/fpsyt.2022.938103 (PMC9719972; doi:10.3389/fpsyt.2022.938103)
Supplement: Supplementary Table S2 — SN ROI. DMN, default mode network; ROI, region of interest; MNI, Montreal Neurological Institute. [file Table_2.docx]

| SaN ROIs | | Main area | MNI coordinates（X，Y，Z） | | |
| --- | --- | --- | --- | --- | --- |
| Anterior SN | ROI1 | Left middle frontal gyrus | -32 | 47 | 20 |
|  | ROI2 | Left insula | -42 | 13 | -5 |
|  | ROI3 | supplementary motor area | 0 | 15 | 44 |
|  | ROI4 | Right middle frontal gyrus | 28 | 44 | 26 |
|  | ROI5 | Right insula | 44 | 14 | -1 |
|  | ROI6 | Left cerebellum | -32 | -58 | -32 |
|  | ROI7 | Right cerebellum | 36 | -57 | -34 |
| Posterior SN | ROI8 | Left middle frontal gyrus | -38 | 36 | 27 |
|  | ROI9 | Left inferior parietal lobule | -57 | -38 | 36 |
|  | ROI10 | Left precuneus | -6 | -51 | 59 |
|  | ROI11 | Right middle cingulate gyrus | 14 | -27 | 44 |
|  | ROI12 | Right lobulus parietalis superior | 22 | -48 | 68 |
|  | ROI13 | Right supramarginal gyrus | 60 | -33 | 33 |
|  | ROI14 | Left thalamus | -12 | -21 | 4 |
|  | ROI15 | Left cerebellum | -35 | -39 | -39 |
|  | ROI16 | Left insula | -37 | -14 | -6 |
|  | ROI17 | Right thalamus | 13 | -13 | 8 |
|  | ROI18 | Right cerebellum | 37 | -42 | -41 |
|  | ROI19 | Right insula | 43 | -4 | -9 |

**Table S2. SN ROI**

*Note: SN, Salience network; ROI, Region of interest; MNI, Montreal Neurological Institute.*
